# Optimally controlling the human connectome: the role of network topology

**DOI:** 10.1038/srep30770

**Published:** 2016-07-29

**Authors:** Richard F. Betzel, Shi Gu, John D. Medaglia, Fabio Pasqualetti, Danielle S. Bassett

**Affiliations:** 1 Department of Bioengineering, University of Pennsylvania, Philadelphia, PA, 19104, USA; 2 Department of Psychology, University of Pennsylvania, Philadelphia, PA, 19104, USA; 3 Department of Mechanical Engineering, University of California, Riverside, Riverside, CA, 92521, USA; 4 Department of Electrical and Systems Engineering, University of Pennsylvania, Philadelphia, PA, 19104, USA

## Abstract

To meet ongoing cognitive demands, the human brain must seamlessly transition from one brain state to another, in the process drawing on different cognitive systems. How does the brain’s network of anatomical connections help facilitate such transitions? Which features of this network contribute to making one transition easy and another transition difficult? Here, we address these questions using network control theory. We calculate the optimal input signals to drive the brain to and from states dominated by different cognitive systems. The input signals allow us to assess the contributions made by different brain regions. We show that such contributions, which we measure as energy, are correlated with regions’ weighted degrees. We also show that the network communicability, a measure of direct and indirect connectedness between brain regions, predicts the extent to which brain regions compensate when input to another region is suppressed. Finally, we identify optimal states in which the brain should start (and finish) in order to minimize transition energy. We show that the optimal target states display high activity in hub regions, implicating the brain’s rich club. Furthermore, when rich club organization is destroyed, the energy cost associated with state transitions increases significantly, demonstrating that it is the richness of brain regions that makes them ideal targets.

One of the goals of modern biology is to understand how a system’s form influences its function. Human brain networks manifest structure-function relationships, with converging evidence suggesting that the brain’s network of white-matter fiber pathways (*structural connectivity*; SC) constrains the intrinsic functional interactions among brain regions at rest (*functional connectivity*; FC), thereby shaping the emergence of coherent spatiotemporal patterns of neural activity^1^,^2^,^3^,^4^,^5^. The effect of these constraints is that over long periods of time (hours, days) resting FC largely recapitulates the underlying SC^6^ so that the strongest functional interactions are often mediated by direct anatomical projections^7^. Over shorter timescales, however, FC is more variable, decoupling from the underlying anatomy to engage specific cognitive systems both at rest^8^,^9^,^10^ and in order to meet ongoing cognitive demands^11^,^12^,^13^.

How does the brain smoothly transition from the activation of one cognitive system to the activation of another? What are the anatomical and topological substrates that facilitate such transitions? One approach for addressing these and similar questions is to model the human brain as a dynamical system, treating brain regions as dynamic elements with time-dependent internal states. As the system evolves, each brain region’s state is updated according to its own history and the states of its connected neighbors. Such models vary in their complexity and neurophysiological basis, ranging from biophysically plausible descriptions of interacting neuronal populations^14^,^15^,^16^,^17^,^18^ to abstract models based on oscillations, diffusion, and epidemic spreading^19^,^20^,^21^,^22^.

In most applications, the question of how to control distributed brain dynamics is not explicitly considered. Here, control refers to the possibility of manipulating a dynamical system so that it evolves to follow a particular trajectory through its state space. We posit that the nature of brain state transitions can be meaningfully addressed with network control theory, which offers a mathematical framework for studying and, ultimately, controlling the evolution of dynamical systems on networks^23^,^24^.

Most dynamical systems can be framed in a control perspective by introducing exogenous input to the system through a set of control sites (network nodes) in the form of time-varying signals. The effect of such inputs is to drive the system along a trajectory through its state space; different inputs, then, result in different trajectories^24^,^25^. The effect of input on a system’s trajectory depends upon (i) the system’s dynamics, (ii) the composition of the control sites, and (iii) the configuration of the system’s nodes and edges into a network (its topology)^24^. Understanding how control occurs in the brain and how these factors contribute to enacting control on the brain is of critical importance, with clear clinical and engineering implications. For example, the efficacy of implantable neuromodulatory devices for suppressing Parkinsonian symptoms^26^, seizure abatement in epilepsy^27^, and other methods for manipulating brain activity, such as transcranial magnetic stimulation^28^, depend on our ability to modify a network’s function by introducing external electromagnetic signals.

In the current work we use network control theory to identify minimum energy input signals that cause the system to transition to and from specific brain states. The input signals are introduced through control sites–brain regions–and can be thought of in two complementary ways. One view is that control signals are issued directly from brain regions, themselves acting as local computational elements administering control over the network. Alternatively, control signals can be viewed as having extracranial provenance, originating from implanted electrodes or other neuromodulatry devices and thereby acting on specific brain regions. In either case, a region’s local contribution can be modeled as an input of energy over time and interpreted as a measure of the amount of effort it puts forth during a control task^25^,^29^.

We seek to better understand the role of brain network topology in determining a region’s energy–what topological factors contribute to making transitions between different brain states more or less effortful? Previous investigations addressing similar questions have focused on how the underlying networks’ statistical properties (e.g. the shape of the degree distribution) and global metrics (e.g. modularity, clustering, etc.) influence the minimum number of control sites necessary to render the network controllable^24^,^30^,^31^,^32^,^33^. Such approaches, while illuminating, are limited. First, the focus on global network statistics makes it difficult to assess the contributions of individual nodes or edges. Second, the classification of networks as either “controllable” or “uncontrollable” overlooks finer gradations in the amount of energy required for control. Though some node-level metrics have been proposed^34^, the precise roles of individual nodes and other topological features in facilitating control is not well understood. Previous applications of network control theory to brain networks investigated related questions, by studying *all possible state transitions* and assuming an infinite time horizon^18^,^35^. Here, we focus on finite-time transitions between a limited set of accessible states, which we choose to correspond to previously-defined brain systems^36^.

The remainder of this report is divided into two sections. In the first, we explore a set of progressively more difficult control tasks, demonstrating that a brain region’s weighted degree (strength) is highly correlated with its control energy, suggesting that regions with many, strong connections contribute disproportionately more energy than regions with few or weak connections. We also study the effect of suppressing input to select sets of brain regions, which reveals a network of compensatory interactions among brain regions. We show that the degree to which one brain region compensates for the removal of another can be predicted with the network measure “communicability”, which measures the strength of direct and indirect pathways between network nodes.

The second section builds on the results from the first. Rather than use a pre-defined set of initial and target states, we objectively assign nodes to initial and target states so as to minimize the cost of the transitions among these states. We find that the optimal assignments implicate highly connected brain regions as ideal targets and weakly connected regions as ideal observers (i.e. they play no role in control). We also show that the energy associated with these optimal assignments is less than what would be expected given a degree-preserving random network model. Finally, we show that when the network’s rich club^37^ is disrupted the energy increases, further suggesting that the configuration of connections among hub regions supports low-energy transitions.

## Mathematical model

We studied a dynamical system in which the brain’s network of white matter fiber tracts among brain regions constrained the following linear time-invariant nodal dynamics^35^,^38^:


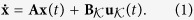


**Figure 1:**
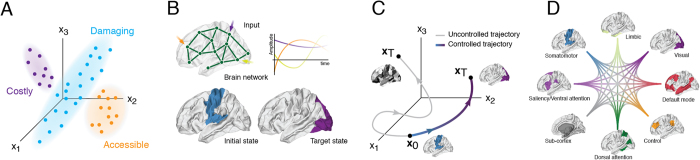
Schematic illustrating structural controllability framework. (**A**) The space of all possible states includes regions that require prohibitively large energy to access as well as damaging configurations. Our analyses are restricted to the accessible region of state space populated by viable configurations. (**B**) A set of time-varying inputs are injected into the system at different control points (nodes; brain regions). The aim is to drive the system from some particular initial state to a target state (e.g. from somatosensory to visual system). (**C**) Example trajectory through state space. Without external input (control signals) the system’s passive dynamics leads to a state where random brain regions are more active than others; with input the system is driven into the desired target state. (**D**) Schematic illustration of all possible system-to-system transitions.

Here, 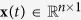, is the state vector whose element, *x*_*i*_(*t*), represents the state (activity level) of brain region *i* at time *t*. The matrix 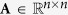 is the symmetric and weighted adjacency matrix, whose element *A*_*ij*_ is the number of detected tracts between regions *i* and *j* normalized by the sum of their volumes. The input matrix, 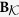, specifies the set of control points, 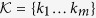, such that:


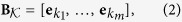


where 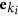 is the *i*th canonical column vector of dimension *n*. The time-varying input signals are denoted as 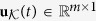 where 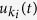 gives the input at control point *k*_*i*_ at time *t* (Fig. 1).

We were interested in the control task where the system transitions from some initial state, **x**_0_ = **x**(*t* = 0), to some target state, **x**_*T*_ = **x**(*t* = *T*). We solved this task using an optimal control framework, deriving the set of minimum-energy inputs, 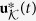, for accomplishing this task (Supporting Information). Each control point’s energy was defined as 
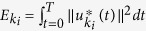
 and the total energy was given by 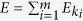.

Rather than investigate all possible transitions, we considered a limited repertoire, focusing on a set of eight states based on systems previously identified in intrinsic functional connectivity studies^36^ (Materials and Methods, Cognitive systems). We analyzed all system-to-system transitions (excluding self-transitions), resulting in 56 possible control tasks. Importantly, because our dynamical model is linear, any possible transition can be written as a linear combination of these transitions (though the resulting transition may not be optimal, in terms of minimum energy). Thus, our results are generally relevant to all transitions.

For a given control task we classified brain regions based on their states at times *t* = 0 and *t* = *T*:

- The *initial* class includes all regions active at *t* = 0 and inactive at *t* = *T* (denoted as **x**_0_).
- The *target* class included all regions inactive at *t* = 0 but active at *t* = *T* (denoted as **x**_*T*_).
- The *bulk* class included all regions inactive at both *t* = 0 and *t* = *T*.

In principal, there is a fourth class of regions active at both *t* = 0 and *t* = *T*. However, given the control tasks we consider here, this case never arises. In subsequent sections we will present our results within this classification scheme.

## Results

### Predicting energy from topology

**Figure 2:**
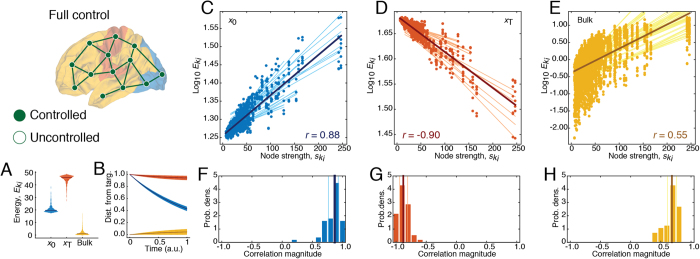
Energy depends on class and strength when all regions are controlled. (**A**) Violin plot of node-level control energies aggregated across all control tasks and divided into initial (**x**_0_), target (**x**_*T*_), and bulk classes. (**B**) Average distance of **x**(*t*) from target state, **x**_*T*_, under free evolution (i.e. no input signals) as a function of time and averaged within each class. (**C**–**E**) Scatterplots of node-level control energies, 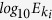, *versus* strengths, 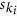, (total normalized streamline counts) across all control tasks. Light lines represent linear regression lines fit to specific control tasks; dark lines represent average regression line. (**F**–**H**) Distributions of Pearson’s correlation coefficients for best-fit lines shown in panels (**C**–**E**).

One of the principal aims of this report was to determine what features of the brain’s topology influence control energy. In this section we assessed the role of topology in determining the energy associated with each of the 56 control tasks. We began this investigation with a simple scenario in which all brain regions served as control points. We observed that 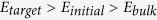 (one-way ANOVA comparing log-transformed energies across all control tasks and participants yielded minimum omnibus test-statistic of *F*(2, 126) = 83.0, and a maximum *p*-value of *p* = 1.0 × 10^−23^; pairwise *t*-tests for *E*_*target*_ > *E*_*initial*_ and *E*_*initial*_ > *E*_*bulk*_ yielded minimum *t*-statistic of *t* = 8.31 and maximum *p*-value of *p* = 1.6 × 10^−9^, Bonferroni adjusted) (Fig. 2A).

To explain this ranking of energies, we need to consider the system’s dynamics under free evolution–the absence of input. In such a case, the system evolves as **x**(*t*) = *e*^**A***t*^**x**_0_, which we obtained by solving Eq. 1. The vector **v**(*t*) = **x**_*T*_ − **x**(*t*), then, specified the distance, *v*_*i*_(*t*), of each region from its target state at time *t*. Intuitively, as distance increased, more energy was required to drive the system towards its desired configuration (Figure S1). Moreover, distance followed a class-specific trajectory (Fig. 2B). At the control horizon, *t* = 1, bulk regions were nearest their target state, followed by initially active regions, followed by target regions, explaining why specific classes required more or less energy.

The previous analysis demonstrated that, foremost, regional control energy depended on class. Within each class, however, we found that much of the remaining variance could be accounted for by regions’ weighted degrees (*strength*; 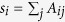). Across participants and control tasks, the logarithm of control energy for regions assigned to initial and bulk classes was both positively correlated with the logarithm of strength (median(interquartile range) correlations of *r* = 0.85(15) and *r* = 0.79(15), respectively) while the opposite was true for target regions *r* = −0.84(13)) (Fig. 2C–E; Figure S2 for summary across all participants). Collectively, these results imply that predicting a brain region’s energy contribution requires both topology and contextualizing a region’s role – i.e. initial, target, bulk–in a given control task.

### Simulated suppression and compensatory effects

In the previous section we investigated a scenario in which all brain regions served as control points, making it possible to control any region’s state directly. In the current section we imagined a more difficult scenario in which the system performed the same control tasks but with specific subsets of brain regions excluded from the control set. These excluded regions, then, could only be manipulated via indirect input from their neighbors–they required help from the rest of the network to achieve their desired state. This framework, which we refer to as “simulated suppression”, is analogous to inhibitory neuromodulation, which can be achieved externally using transcranial magnetic stimulation (TMS), in which a brain region’s local activity is suppressed but still receives inputs from surrounding areas^39^,^40^. This notion of simulated supppresion could also be achieved internally via competitive or inhibitory dynamics among neuronal populations.

Using simulated suppression we investigated how the suppression of input to specific regions changed the energies of the remaining, unsuppressed, regions. We interpreted such changes as a measure of compensation. In this section, we explored a series of progressively more difficult control scenarios in which we suppressed both individual brain regions and entire classes of regions.

#### Suppression of individual brain regions

**Figure 3:**
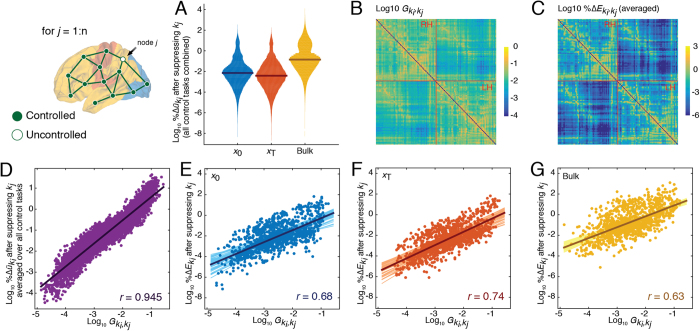
Communicability predicts compensation (or change in energy) when individual regions are suppressed. (**A**) Violin plot of percent change in regions’ energies (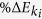) after suppressing region *k*_*j*_ aggregated by class. (**B**) Compensation network in matrix form; the weights of the compensation network are defined as the percent change in energy of region *k*_*i*_ (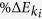) after suppressing region *k*_*j*_ averaged across all control tasks. (**C**) Weighted communicability matrix, which measures the total strength of direct and indirect connections between two regions. (**D**) Scatterplot showing the correlation of communicability and the compensation network weights. (**E**–**G**) Percent change in the energy of region *k*_*i*_ after suppressing region *k*_*j*_. Each class is shown separately – **x**_0_ are active at time *t* = 0, **x**_*T*_ are active at time *t* = T, and bulk nodes are inactive at both *t* = 0 and *t* = *T*. Matrices in panels (**B**,**C**) are shown with logarithmic scaling.

In this section we suppressed individual brain regions, one at a time, and repeated the same control tasks as the previous section. We expected that with fewer control points the total control energy, *E*, would increase. We found that this was largely the case, with the greatest percent changes in energy occurring among bulk regions (Fig. 3A).

The simulated suppression framework allowed us to calculate the extent to which brain regions engaged in compensatory relationships with one another, wherein the suppression of one region consistently resulted in increased energy of another region. We were also interested in determining to what extent these compensatory relationships could be predicted based on topological properties of the network. We hypothesized that the strength of compensatory relationships should depend upon the extent to which they were interconnected. We further reasoned that even indirectly-connected regions should be able to compensate for one another provided that they were linked by many multi-step paths. This intuition can be formalized as the *communicability* between two regions, a statistic that quantifies the strength of both direct and indirect pathways between node pairs^41^. Communicability can be thought of as the capacity for two regions to communicate with one another by pathways of all topological lengths (Fig. 3B) (Materials and Methods, Network communicability).

For each control task we calculated the percent change in energy of region *k*_*i*_ after suppressing region *k*_*j*_, which we denote as 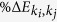 (Fig. 3C). When averaged across all control tasks we found excellent correspondence between this measure and weighted communicability, with a correlation of *r* = 0.95(0.01) across participants (Fig. 3D). The same relationship persisted (albeit attenuated) when we examined specific control tasks and sub-divided regions according to their class: for initial *r* = 0.74(0.08), for target *r* = 0.80(0.06), and for bulk *r* = 0.68(0.11)) (Fig. 3E–G). These results suggest that both direct and indirect connections play important roles in compensating for suppressed brain regions (Figure S3 for summary across all participants).

#### Suppressing initial *or* target classes

**Figure 4:**
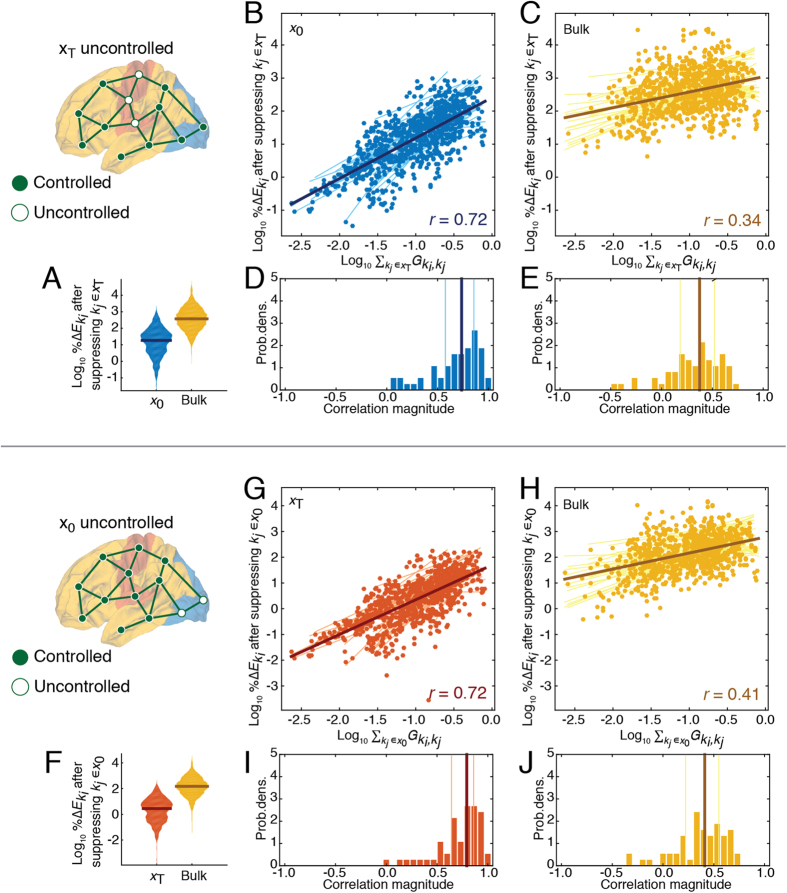
Summary of initial or target class suppression. (**A**) Violin plot showing percent changes in energy 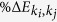, for the remaining nodes after excluding all target nodes from the control set. (**B**,**C**) Scatterplot of the percent change of region *k*_*i*_’s energy after suppressing all target nodes against *k*_*i*_’s communicability to all target nodes. (**D**,**E**) Distributions of Pearson’s correlation coefficients between change in energy and communicability, 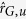, for initial and bulk nodes. Panels (**F**–**J**) recapitulate (**A**–**E**) but with initial nodes excluded from the control set rather than target nodes.

We extended the simulated suppression framework by simultaneously suppressing entire classes of brain regions, resulting in considerably more difficult control tasks. In particular, we suppressed all regions assigned to either initial or target classes. As in the previous section, we found that bulk regions exhibited the greatest percent change in their energies (Fig. 4A,F). Also in agreement with the previous section, we found that the percent change in a region’s energy was predicted by its total communicability to the suppressed class (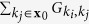 and 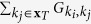) (Fig. 4B,C,G,H). When the target class was suppressed, the correlation of communicability and change in energy was *r* = 0.76(0.24) and *r* = 0.38(0.36) for the remaining initial and bulk regions, respectively. When the initial class was suppressed, the correlation of communicability and change in energy was *r* = 72(0.30) and *r* = 0.32(0.41) for the remaining target and bulk regions (Figure S4 for summary across all participants).

#### Suppressing initial *and* target classes

**Figure 5:**
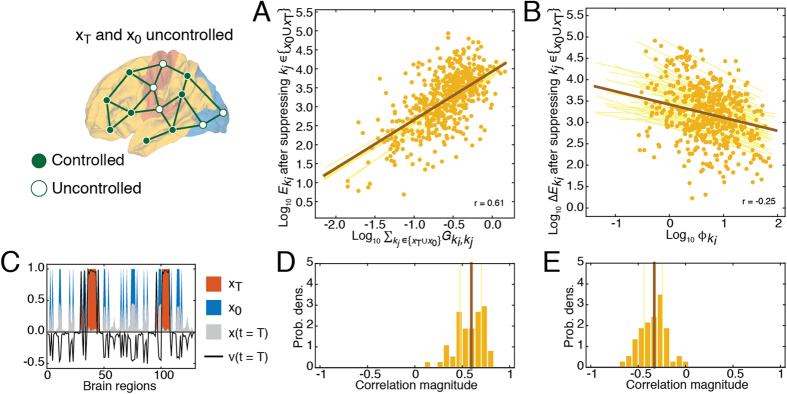
Summary of initial and target class suppression. (**A**) Only bulk nodes are directly controlled. Their energies, 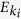, are strongly correlated with their communicability to the initial and target nodes, 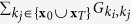. (**B**) The percent change in total energy going from the *full control* experiment to the *no control* experiment was negatively correlated with 
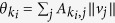
, or the weighted distance of controller *k*_*i*_’s neighbors from their respective target states relative to their states under free evolution. (**C**) An illustrative example of the components that go into calculating 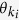. The blue and orange bars represent the state of the system at times *t* = 0 and *t* = *T*. The blue bars indicate the activation of DMN nodes, in this case, while the orange bars indicate activation of VIS nodes. The grey bars represent the state of the system at time *t* = *T* under free evolution after starting in **x**_0_ = *DMN*. Finally, the black line is equal to **v**(*t*) = **x**_*T*_ − **x**_0_, or the element-wise difference between the orange and grey bars. (**D**,**E**) Distributions of Pearson’s correlation coefficients from the best-fit lines shown in panels (**A**,**B**), respectively.

Finally, we explored the consequences of simultaneously suppressing both initial and target regions, leaving only the bulk as control points. In the previous sections if suppression was applied to initial or target nodes, the remaining bulk could perform the same control task as the missing nodes. Here, however the bulk was tasked with performing two duties: simultaneously turning on and off target and initial regions, respectively. The most energetic members of the bulk were those with high levels of communicability to either initial or target regions, thereby affording them the possibility of directly and indirectly controlling the states of those classes (Fig. 5A) (across participants, the correlation of 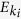 and 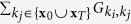, the communicability of region *k*_*i*_ to both initial and target regions, was *r* = 0.57(0.25)). These results further suggest that bulk regions–those not actively changing their own state during a control task–nonetheless acted as compensators when other regions become compromised.

We also were able to predict bulk regions whose energy contributions increased by the greatest amount. As noted earlier, the vector **v**(*t*) = **x**_*T*_ − *e*^**A***t*^**x**_0_ gives the Euclidean distance in nodes’ states at time *t* with their respective target states. We hypothesized that the greater the distance a bulk region’s neighbors were from their target states would be related to how much additional energy that region would have to contribute. To this end, we calculated 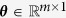 whose element 
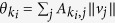
 gives the total distance of *k*_*i*_’s neighbors from their target states weighted by the strength of *k*_*i*_’s connection to those nodes. We found that the logarithm of 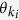 and 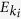 were robustly negatively correlated across both participants and control tasks (*r* = −0.33(0.18)) (Fig. 5B) (Figure S5 for summary across all participants).

Collectively, the results reported in this section make two important points. First, bulk regions–those not changing their state from active to inactive (or *vice versa*)–exhibited the greatest increase in energy following the suppression of other regions, suggesting that these “bystanders” may play important compensatory roles in the control of brain dynamics. Secondly, in demonstrating that brain region’s compensatory relationships are correlated with their communicability, we implicate indirect connections as important pathways through which compensatory relationships emerge.

### Optimal class assignments

The human brain has evolved to exhibit many near-optimal features. For example, drives to reduce total wiring length^42^,^43^ and metabolic expenditure^44^,^45^ are thought to impose powerful constraints on the brain’s organization, development, and growth. In addition to costs associated with architecture and energy, it has been suggested that the brain is capable of implementing computational algorithms for minimizing arbitrary cost functions^46^. In this section, we ask the question: *if* the brain were capable of implementing an optimal control algorithm and wished to minimize total energy expenditure, which brain regions would it choose as its initial and target states? One approach for answering this question would be to enumerate all possible divisions of brain regions into the three aforementioned classes and then to apply optimal control to determine the total transition energy. The optimal class assignments, then, would be the division associated with the smallest transition energy. This approach, however, is computationally intractable due to the prohibitively large number of possible divisions. An attractive alternative is to re-state this question in terms of an optimization problem and to use some heuristic for estimating the optimal division. In this section we investigated such an approach. Specifically, we identified class assignments that minimized the objective function, 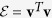, where **v** is defined in the same way as before. We chose this particular objective function because it is highly correlated with control energy (Figure S1) and also for computational ease, as it can be calculated in a more straightforward manner than energy, which requires first deriving the optimal control signals.

We used a simulated annealing algorithm to minimize 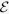 and generate estimates of the probability with which each brain region was assigned to initial, target, and bulk classes. Across participants we found that the probability of a region being assigned to either the target and bulk classes was highly correlated with its weighted degree (*r*_*target*_ = 0.88(0.03) and *r*_*bulk*_ = −0.73(0.07); maximum *p*-value of *p* = 2.5 × 10^−16^), while the nodes assigned to the initial class were not closely associated with weighted degree (*r* = −0.02(0.09); minimum *p*-value of *p* = 0.27). These results suggest that highly and weakly connected regions are ideal targets and bystanders, respectively.

What aspects of the network’s organization determines class assignments? One possibility is that the low-energy transitions facilitated by these assignments are merely a consequence of the number and weight of connections a brain region makes–i.e. they do not depend on the actual configuration of a network’s connections. To test this hypothesis, we randomized connection placement while preserving the number of connections that each brain region makes (generating 100 random networks for each participant) and evaluated the objective function for the optimal class assignments given these networks. We observed that across all class compositions the randomized networks were associated with significantly greater energies than the observed networks (non-parametric test, max *p* < 1 × 10^−15^).

### Rich club promotes low-energy transitions

**Figure 6:**
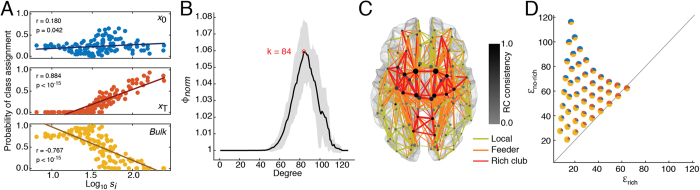
Rich club detection and optimal class assignments. (**A**) Class assignment probabilities as a function of the logarithm of each brain region’s strength. (**B**) Normalized rich club coefficient as a function of degree. Gray envelope represents ± one standard deviation. (**C**) Topographic distribution of rich club nodes and classification of edges (top 5% for visualization) as either “local” (links two non-rich club regions), “feeder” (links a rich club region to a non-rich club region), or “rich club” (links a rich club region to another rich club region). The size of each node is proportional to its degree and the darkness of each point indicates the likelihood, across participants, that that node was assigned to the *k* = 84 rich club. (**D**) Comparison of energy with rich club intact (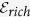) versus rewired rich club (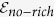). Each point (pie chart) represents a particular composition of class assignments. The gray line indicates the “break-even” line–points along the line correspond to optimal class assignments that have approximately equal average energy both with and without a rich club. Points above this line indicate that rewiring the rich club leads to increased energy associated with the optimal class assignments.

The observation that highly-connected brain regions make good targets and poor bystanders suggests that hub regions and, perhaps, rich clubs play an important role in facilitating low-energy control^37^,^47^. Intuitively, a rich club is a collection of hubs–highly connected, highly central regions–that are more densely interconnected to one another than expected. This type of organization is thought to promote rapid transmission and integration of information among brain regions^48^. Indeed, rich club regions were more likely to be assigned to the optimal target state (Figure S6). The previous null model, wherein all connections were randomized, tested the null hypothesis that structureless networks could produce comparable levels of energy. To test whether a network’s rich club influences energy requires a more subtle and specific null model. Moreover, a network’s rich club is a pseudo-continuous structure and can be defined at multiple resolutions. Our focus was on the neighborhood of binary rich clubs identified at *k* = 84–i.e. all brain regions included in the rich club must have degree of at least 84–which corresponds to the maximum normalized rich club coefficient obtained across all participants (Fig. 6A). At this level, the most consistent rich club regions across participants included subcortical regions thalamus, caudate, putamem and hippocampus as well as precuneus, isthmus cingulate, posterior cingulate, lateral orbito-frontal, and insular cortex. These regions are in close agreement with previously-described rich clubs and hubs^1^,^37^,^47^,^49^,^50^ (This particular rich-club composition is consistent within a neighborhood of *k* = 84; Figure S7).

We implemented a null model where we rewired only connections among rich club members (while preserving degree) so that the density of connections among rich club members was as low as possible. We observed that as long as eight brain regions (the smallest increment that we considered) were assigned as initially-active regions, then rewiring connections to dissolve the rich club alway yields increased energy (non-parametric test, max *p* < 1 × 10^−15^), suggesting that the brain’s rich club, specifically, supports low-energy transitions from a diverse set of initial states to a target state of high-strength hub regions (we verify that this result holds for rich clubs defined at *k* = 80 to *k* = 88; Figure S8).

## Discussion

In this report we used network control theory to investigate the role of the brain’s anatomical network in supporting transitions among different brain systems. In the first section we focused on transitions among a pre-defined set of brain systems and demonstrated, in agreement with earlier work, that brain regions with many strong connections were associated with increased energies. We further demonstrated that when the control set is perturbed, by suppressing control of both individual brain regions and entire classes, the remaining regions compensate for the loss by increasing their own energies. Moreover, we showed that the percent change in a controller’s energy could be predicted by its communicability to the suppressed regions, highlighting the role of indirect communication paths. In the second section, we sought to objectively identify initial and target states that could be transitioned to and from for little energy. We found that the optimal initial states were diverse while the probability that a region was among the target state was highly correlated with its weighted degree. We showed that transitions among these optimal class assignments were, in part, facilitated by the brain’s rich club; when connections among rich club regions were rewired, the energy associated with such transitions consistently increased.

### From descriptive to predictive network models

The study of networked neural systems has advanced rapidly in the past decade. While early analyses focused on the topological properties of SC networks such as their small-worldness^51^,^52^ or the presence of hubs, rich clubs, and modules^1^,^37^, the focus of recent work has shifted from static descriptions to dynamical systems models, making it possible to investigate how network topology shapes passive dynamics (i.e. no inputs)^19^,^20^,^21^,^22^. A natural extension of these and other recent studies is to incorporate exogenous input into the dynamical model. Such an extension makes it possible to begin addressing questions related to the control of the brain. At the level of large-scale human brain networks, these theoretical questions are only now beginning to be addressed^18^,^35^, though the utility of this approach is obvious, showing promise in stimulation-based treatment of epilepsy^27^. These first studies offered statistics for characterizing the extent to which brain regions contribute to making the entire state space accessible for a network–i.e. rendering it controllable. The entire state space, however, likely contains states that, for one reason or another, should actively be avoided by the system. In this present study we sought to characterize the energy contributions of brain regions based on transitions among a limited state space populated by seemingly neurophysiologically plausible states.

### Structural predictors of the ease or difficulty of control

The aim of this report was to shed light on the features of a network’s topology that contribute to making a control task easier or more difficult. Toward this end we made a number of contributions and novel observations. First, we presented a classification system of nodes for studying specific control tasks. We showed that for the control tasks we investigated, a node’s control energy is highly correlated with its strength. The nature of this relationship, however, depends critically on whether a node is classified as part of the initial, target, or bulk set–the energies of nodes that are initially active and later inactive (initial) as well as nodes that, at the boundary conditions of *t* = 0 and *t* = *T*, are inactive (bulk) maintain strong positive relationships with node strength; the opposite is true for target nodes, which are negatively correlated with node strength. In other words, to predict a brain region’s control energy we require both its context – its role within a given control task – and its embedding within the network’s topology.

Under this framework, we explored a series of progressively more difficult control tasks in which we suppressed specific subsets of nodes. This set of experiments highlighted the brain’s compensation network–a network whose edge weights represent the percent change in a node’s energy when other nodes are excluded from the control set. We went on to show that *communicability* between two nodes was highly correlated with their compensation weight, which is interesting for several reasons. Communicability measures the strength of direct and indirect connections between two nodes^53^, which suggests a possible functional role for multi-step pathways in the human brain^5^. Namely, that when a brain region’s capacity for control is compromised–e.g. acute ischemic stroke or electro-magnetic simulation–the regions that “pick up the slack” and take on expanded control roles include, as one might anticipate, those with direct connections to the compromised region, but also those with many indirect and potentially long-distance connections. This observation serves as a potential mechanistic account of diaschiatic phenomena, where the effect of a focal lesion on brain function is observed at some nontrivial distance from the lesion site^54^,^55^,^56^. Additionally, this observation is in line with recent work showing that including multi-step pathways in predictive models of FC leads to decreased error rates and improved predictions of resting state functional connectivity^5^,^7^.

Interestingly, we observed that bulk regions exhibited the greatest percent increase in their energy. This suggests that regions not directly involved in a particular control task actually play a disproportionately greater compensatory role than those directly involved–i.e. initial and target regions. Intuitively, these results may provide a context to understand cognitive dysfunction observed in neurological conditions that involve region damage or loss, such as traumatic brain injury or Alzheimer’s disease. From a control theoretic perspective, as brain regions suffer damage, the increased burden to bulk nodes for performing multiple control tasks may interfere with one another. Bulk regions may become sites of processing interference due to competition between new compensatory roles and older noncompensatory roles. This increased competition between control tasks could have implications for the consequences of brain injury and neurodegeneration over longer timescales^57^. In particular, increased processing burdens may result in later deleterious effects on regions that assume a disproportionate share of compensatory burdens^58^, resulting in a cascade of later failures across the brain.

Regional suppression is also relevant to models of conflict processing and the cognitive effort expended in doing so^59^. In such models, computational executive control mechanisms can be deployed for a limited number of simultaneous tasks, resulting in opportunity costs. With increased opportunity costs comes the phenomenology of effort (motivation, fatigue, boredom), which, in turn, alters the control mechanisms. In the context of our control theoretic view of task control, as node suppression introduces potential conflicts between control tasks among bulk regions, we might expect increases in perceived effort, difficulty managing sustained tasks or task switches, and negative consequences for task performance in a variety of disorders and tasks with control demands. Future studies could test this prediction by mapping the cognitive difficulties and perceived effort that arise from conflicting control demands following real region damage or following non-invasive temporary suppression. Specifically, we would expect that cognitive performance would be low and perceived effort high in general in individuals where a relatively small set of bulk regions assume a relatively high set of compensatory roles across control tasks. Thus, our control theoretic approach may provide a practical means to quantify effort and processing conflicts in health and disease.

### The rich club as a control backbone

Whereas we have approached control from an engineering perspective, in cognitive neuroscience the term control refers to a set of processes, which include memory and attentional systems, for guiding behavior towards a particular goal^60^,^61^. Control is thought to be instantiated via an anatomical substrate consisting of cortical^62^ and sub-cortical areas^62^ acting largely through inhibitory mechanisms. An important question concerns how this neuro-mechanistic account of control interfaces with current theories on the role of brain connectivity in normative brain function and to what extent it is related to the engineering-focused approach adopted here. In the second section we identified brain regions that act, in an objective sense as optimal initial, target, and bulk classes for control. We showed that the probability of a region being classified as target or bulk was closely related to its strength, with high and low strength regions more likely to be classified as targets and bulk, respectively. The initial class, on the other hand, had a more diverse constituency and was not obviously related to strength. We went on to show that the energies associated with these class assignments were much lower than those obtained from degree-preserved random networks, indicating that the class assignments were driven by some non-trivial aspect of the network’s topology. We further showed that when the brain’s rich club was dissolved the energy associated with these optimal assignments increased, suggesting that the rich club contributes in facilitating low-energy transitions from a diverse set of initial states to target states composed of high-strength, high-degree rich club regions.

The rich club is typically thought of as an integrative backbone that allows hub regions to communicate with one another, facilitating rapid communication and transmission of signals across the entire brain^48^,^63^,^64^. Despite this supposition, there is some controversy as to the rich club’s precise role in network communication, with some indication that rich club hubs are primarily drivers of network dynamics–acting in a top-down fashion to influence different sub-systems^65^,^66^,^67^. On the other hand, rich club regions are highly connected to the rest of the network and, by this virtue, are not only in a position to influence their neighbors, but also to be influenced *by* their neighbors. Indeed, computational models suggest that rich clubs adopt stable, regular behavior as a consequence of receiving input from a multitude of sources^68^. Our results agree with this latter account in some respects. We observed that high-degree, rich club regions are best suited for roles as targets that can be transitioned into from a diverse set of initial states. Part of why rich club regions are so successful in this respect is by virtue of their many connections–no matter where input is injected into the system, there is a high probability that the signal will propagate from its source to activate regions in the rich club, suggesting a possible explanation for why rich club regions, which overlap considerably with the brain’s default mode network, tend to be active in the default mode of resting state function^67^. More relevant to the present discussion, this also suggests that under certain conditions the rich club (or at least highly connected regions) may be in a better position to be indirectly controlled than weakly connected regions on the network periphery.

### Methodological considerations

Several important methodological considerations are pertinent to this work. First, we relied on diffusion spectrum imaging and tractography to infer the presence of large-scale fiber tracts in participants’ brains. These methodologies are imperfect and can detect spurious tracts or fail to detect existing tracts^69^,^70^. However, at present there exist no other non-invasive methods for reconstructing human structural connectivity networks. Future work will likely address these shortcomings by introducing improved tractography algorithms and imaging techniques^71^.

Another important consideration is our use of a linear dynamical model despite the fact that, by most accounts, brain activity is fundamentally non-linear^6^. Our justification for using such dynamics is twofold. First, the emphasis of this paper is on the role that the brain’s structural connectivity network plays in control. While the form of dynamics certainly contributes to making a system controllable or not, we focus primarily on the contribution of the network’s topology. Secondly, a linear dynamics is in line with other papers that investigate control theory with an emphasis on topology^24^,^29^,^72^. Moreover, describing non-linear systems in terms of a linear approximation in the neighborhood of its equilibrium points is common^73^, and makes it possible to apply linear control to otherwise intractable systems^35^. It should also be noted that even if we wish to proceed using a linear dynamical model, the coupling matrix–here selected to be the brain’s structural connectivity matrix–can be further modified. One possibility is to use a coupling matrix based on the observed pattern of co-fluctuations (correlations) between brain regions’ activity time series^74^. Should such an approach be used in the context of control, the assumptions of the dynamical model would need to be justified carefully, the predictions validated, and the associated statistical methods potentially extended to account for the differences in the mathematical properties of correlation matrices (positive semi-definite) in comparison to structural matrices built on streamline counts. Here we restrain ourselves to building on the justifications, validations, and statistical extensions for structural matrices described in our prior work^35^. We expand on this discussion in the Supplementary Material under the heading *Justification for studying linear dynamics*.

An additional concern is the decision to represent functional systems as non-overlapping–i.e. brain regions can be assigned to one system and one system only. In reality, of course, there is no clear one-to-one mapping of brain regions to cognition and behavior; many brain areas are poly-functional. There is, however, a trend in cognitive neuroscience, and especially network neuroscience, to treat brain regions as non-overlapping (see^36^,^75^,^76^). Following this tradition was practically useful for our analyses, as we took advantage of the non-overlapping nature of systems to assign brain regions uniquely to initial, target, and bulk classes–had we used overlapping systems it would have been possible for regions to appear in both the initial and target classes, for example. Future work should explore techniques for obtaining overlapping clusters (and therefore overlapping network states) and investigating optimal control in this context.

A final limitation is the form of the communicability measure, which (when applied to a binary networks) weighs longer paths exponentially less than shorter paths. While this standard form appears sufficient for our purposes here, alternative weighting schemes may provide additional insight in the role of multi-step paths on network control.

## Conclusion

Understanding how control occurs in the brain, and how we can use external interventions to affect that control, has broad implications across the cognitive and clinical neurosciences. By examining control strategies implemented in finite time and with limited energy, we were able to uncover fundamental principles of brain structure that impact the ease or difficulty of control tasks informed by the systems known to perform diverse cognitive functions. It is intuitively plausible that these principles may be altered by psychiatric disease and neurological disorders, via a change in underlying structural connectivity. In future, it will be interesting to understand how individual differences in brain structure affect individual differences in the natural implementation of control (e.g., cognitive control) and resulting behavior in executive domains. Moreover, it will be interesting to understand how these individual differences might also directly affect susceptibility and response to external interventions via invasive or non-invasive neuromodulation. Such an understanding would provide critical groundwork for personalized medicine.

## Materials and Methods

### Data acquisition and processing

Diffusion spectrum images (DSI) were acquired for a total of 30 subjects along with a T1-weighted anatomical scans. We followed a parallel strategy for data acquisition and construction of streamline adjacency matrices as in previous work^35^. DSI scans sampled 257 directions using a Q5 half-shell acquisition scheme with a maximum *b*-value of 5,000 and an isotropic voxel size of 2.4 mm and an axial acquisition with the following parameters: repetition time (TR) = 5 s, echo time (TE) = 138 ms, 52 slices, field of view (FoV) (231, 231, 125 mm). All procedures were approved in a convened review by the University of Pennsylvania’s Institutional Review Board and were carried out in accordance with the guidelines of the Institutional Review Board/Human Subjects Committee, University of Pennsylvania. All participants volunteered with informed consent in writing prior to data collection.

DSI data were reconstructed in DSI Studio (www.dsi-studio.labsolver.org) using *q*-space diffeomorphic reconstruction (QSDR)^77^. QSDR first reconstructs diffusion-weighted images in native space and computes the quantitative anisotropy (QA) in each voxel and warped to a template QA volume in Montreal Neurological Institute (MNI) space using the statistical parametric mapping (SPM) nonlinear registration algorithm. Once in MNI space, spin density functions were reconstructed with a mean diffusion distance of 1.25 mm using three fiber orientations per voxel. Fiber tracking was performed in DSI studio with an angular cutoff of 55°, step size of 1.0 mm, minimum length of 10 mm, spin density function smoothing of 0.0, maximum length of 400 mm and a QA threshold determined by DWI signal in the colony-stimulating factor. Deterministic fiber tracking using a modified FACT algorithm was performed until 1,000,000 streamlines were reconstructed for each individual.

Anatomical scans were segmented using FreeSurfer59 and parcellated using the connectome mapping toolkit^78^. A parcellation scheme including *n* = 129 regions was registered to the B0 volume from each subject’s DSI data. The B0 to MNI voxel mapping was used to map region labels from native space to MNI coordinates. To extend region labels through the grey-white matter interface, the atlas was dilated by 4 mm^79^. Dilation was accomplished by filling non-labelled voxels with the statistical mode of their neighbors’ labels. In the event of a tie, one of the modes was arbitrarily selected. From these data, we constructed a structural connectivity matrix, **A** whose element *A*_*ij*_ represented the number of streamlines connecting different regions, divided by the sum of volumes for regions *i* and *j*.

### Cognitive systems

The human brain can also be studied as a network of functional connections. Functional connectivity networks are modular, which means that they can be partitioned into non-overlapping sub-systems^36^,^75^,^80^. These sub-systems are referred to as *intrinsic connectivity networks* (ICNs) and have distinct cognitive and behavioral fingerprints^81^,^82^. The ICN definition used here was based on a previously-delineated set of system boundaries^36^, and included default mode (DMN), control (CONT), dorsal attention (DAN), saliency/ventral attention (SAL/VAN), somatomotor (SM), visual (VIS), limbic (LIM), and sub-cortical (SUB) systems. In order to assign each region of interest to a single system, we mapped both atlases to a common surface template (*fsaverage*) and calculated the overlap (number of common vertices) of each region of interest with each of the seven ICNs. A region’s ICN assignment was defined as the system with which it overlapped to the greatest extent (Figure S9).

### Control tasks

We considered transitions from an initial state, **x**_0_, to a target state, **x**_*T*_, where *T* = 1 is the control horizon. Initial and target states were selected to correspond to specific cognitive systems. For example, one possible control task was to start in a state where the default mode network (DMN) was maximally active, and to transition to a state where the visual system (VIS) becomes maximally active. Intuitively, such a transition might correspond to the presentation of a visual stimulus at rest, eliciting activation of visual cortex while suppressing activation of the default mode system. In this context, the control question one asks is which nodes play a role in the minimum energy trajectory between these states. We modeled this control task by starting DMN regions in an active state at *t* = 0:


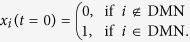


Similarly, when *t* = *T*, only visual regions were in an active state:


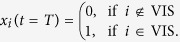


Given these boundary conditions, we calculated the optimal inputs, 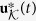 to effect the transition from specified initial state to specified target state. In general, state vectors at other times can take on any real value. Though we considered this limited set of states, the linear model of brain dynamics means that any transition can be written as a linear combination of the transitions we studied here.

### Network communicability

The adjacency matrix, **A**, encodes a network’s direct connections. In addition, communication between pairs of nodes can also take advantage of indirect connections. To quantify the extent to which nodes are connected indirectly, one can calculate the communicability matrix^41^, 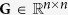, whose element 
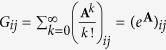
. The element *G*_*ij*_, then, represents the weighted sum of walks of all lengths. The *k*! in the denominator means that longer walks contribute disproportionately less compared to shorter walks. Communicability has been generalized to weighted networks, such as those considered here^83^. Specifically, 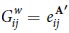, where 
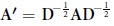
 and 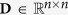 is the square matrix whose diagonal elements 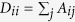. Communicability is also related to the linear dynamics we study here. Solving (1) for the special case where there is no input, the evolution of the system is described by the matrix exponential of the connectivity matrix.

### Optimal class assignments and simulated annealing

In the second section of this paper–*optimal class assignments*–we sought a division of nodes into initial, target, and bulk classes corresponding to the smallest possible control energy. To identify the optimal division, we needed to solve the minimization problem:


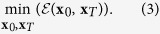


Note that we are not minimizing control energy directly; instead, we minimize 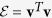 where 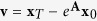, a more computationally tractable measure that is highly correlated with the actual control energy (see Figure S1). This minimization problem cannot be solved analytically and therefore the optimal division of the network into initial, target, and bulk classes can only be approximated. To generate good approximations of the optimal solution, we use the optimization heuristic *simulated annealing* (SA)^84^. SA is a heuristic that can be applied to a wide range of minimization problems. Briefly, SA begins with an initial estimate of the solution (which can be generated randomly) and a temperature parameter. Next, the quality of the initial estimate is calculated according to an objective function (usually supplied by the user), after which the initial estimate of the solution is modified slightly with the addition of a small amount of noise. If the modification results in a higher quality estimate of the solution, then this modification is retained and propagated to the next iteration. Even if the solution results in decreased quality, it is still retained with some probability that decays monotonically as a function of the temperature parameter. This makes it possible, at high temperatures, to sample a range of possible solutions from the parameter space. Gradually, the temperature is reduced, meaning that the only solutions that get retained are those of higher quality. The algorithm continues until some termination criteria are reached–e.g. a pre-specified number of iterations have passed without uncovering a new estimate of the optimal solution.

For our specific minimization problem, we started with a random division of nodes into initial, target, and bulk classes. The proportion of nodes assigned to each of the three classes was fixed from the beginning and did not change over the course of minimization. At each iteration, we propose a new estimate of the optimal solution by randomly selecting two nodes assigned to different classes and swapping their class assignments. If the new estimate reduces 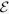 (i.e. is of increased quality), we accept the swap. If the estimate results in a 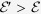, we accept the swap with probability 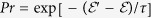. We initialized the algorithm with a temperature *τ*_0_ = 1. In total, the algorithm consisted of 100 stages, each comprised of 5000 iterations. The temperature was fixed throughout each stage and, following each stage, was decreased by a factor of 0.925, so that by the 100th stage, the temperature was 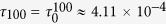. Because we had no prior knowledge of what proportion of nodes should be assigned to initial, target, and bulk classes, we tested 44 possible proportions. These proportions were selected by fixing the number of bulk nodes to be 48, 56, 64, 72, 80, 88, 96, or 104. The nodes were then divided among initial and target classes in multiples of eight. For example, suppose we fixed the number of bulk nodes to be 64. This leaves 64 nodes to be divided among initial/target classes. The possible divisions are: 8/56, 16/48, 24/40, 32/32, 40/24, 48/16, and 56/8. Once the proportion was determined, we ran the SA algorithm 50 times and retained the 100 highest-quality, unique estimates of the optimal division.

### Rich club detection

A rich club refers to a subset of nodes, all of degree ≥*k*, that are densely connected to one another^85^,^86^. The density of any such subset is summarized by its rich club coefficient: 
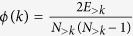
. We calculated *ϕ*(*k*) for all possible values of *k* across all participants and subsequently normalized rich club coefficients by comparing them against the distribution of coefficients obtained from an ensemble of randomized networks (preserved degree sequence)^87^. It is sometimes the case that there is no single rich club–there may be many statistically significant rich clubs spanning a range of *k*^37^. For ease of description, however, we focus on the rich club detected at *k* = 84, which corresponds to the greatest value of *ϕ*_*norm*_ averaged across participants (Fig. 6A). We explore the consistency of this rich club in Figure S6.

## Additional Information

**How to cite this article**: Betzel, R. F. *et al*. Optimally controlling the human connectome: the role of network topology. *Sci. Rep*. **6**, 30770; doi: 10.1038/srep30770 (2016).

## Supplementary Material

Supplementary Information
