# Supplementary material for: Optimally controlling the human connectome: the role of network topology
Source: Sci Rep. 2016 Jul 29;6:30770. doi: 10.1038/srep30770 (PMC4965758; doi:10.1038/srep30770)
Supplement: Supplementary Information [file srep30770-s1.pdf]

Supplement to: Optimally controlling the human connectome: the role of network topology

Richard F. Betzel[1], Shi Gu[1], John D. Medaglia[1,2], Fabio Pasqualetti[3], and Danielle S. Bassett*[1,4]

[1] Department of Bioengineering, University of Pennsylvania, Philadelphia, PA, 19104
[2] Department of Psychology, University of Pennsylvania, Philadelphia, PA, 19104
[3] Department of Mechanical Engineering, University of California, Riverside, Riverside, CA, 92521
[4] Department of Electrical and Systems Engineering, University of Pennsylvania, Philadelphia, PA, 19104

June 16, 2016

This file includes:

1. Derivation of optimal control inputs.
2. Justification for studying linear dynamics.
3. Sensitivity of results to variation of parameters.
4. Robustness to alternative definitions of functional systems.
5. Robustness to alternative parcellations and number of nodes.
6. Supplementary figures S1 - S12.

*dsb @ seas.upenn.edu

1 Optimal Control

There can be an infinite number of inputs, $\mathbf{u}_{\mathcal{K}}$, for driving a system from $\mathbf{x}_0$ to $\mathbf{x}_T$. We wish to find the inputs corresponding to the following minimization problem:

$$\min_{\mathbf{u}} \int_0^T (\mathbf{x}_T - \mathbf{x})^T(\mathbf{x}_T - \mathbf{x}) + \rho \mathbf{u}_{\mathcal{K}}^T \mathbf{u}_{\mathcal{K}}. \tag{1}$$

Here, $\rho \in \mathbb{R}_{>0}$ is a free parameter that scales the relative importance of the first term to the second term in the integral. We set $\rho = 100$. To identify the optimal inputs, $\mathbf{u}_{\mathcal{K}}$, we define the Hamiltonian:

$$H(\mathbf{p}, \mathbf{x}, \mathbf{u}, t) = (\mathbf{x}_T - \mathbf{x})^T(\mathbf{x}_T - \mathbf{x}) + \rho \mathbf{u}_{\mathcal{K}}^T \mathbf{u}_{\mathcal{K}} + \mathbf{p}(\mathbf{A}\mathbf{x} + \mathbf{B}_{\mathcal{K}}\mathbf{u}). \tag{2}$$

In this expression $\mathbf{A}$ is a scaled version of the weighted connectivity matrix. Specifically, we divide the original matrix by its largest eigenvalue and subtract 1 from all diagonal elements. This effectively ensures that all eigenvalues are less than zero and renders the system stable. From the Pontryagin minimization principle, if $\mathbf{u}_{\mathcal{K}}^*$ is an optimal solution to the minimization problem with corresponding trajectory, $\mathbf{x}^*$, then there exists $\mathbf{p}^*$ such that:

$$\frac{\partial H}{\partial \mathbf{x}} = -2(\mathbf{x}_T - \mathbf{x}^*) + \mathbf{A}^T \mathbf{p}^* = \dot{\mathbf{p}}^* \tag{3}$$

$$\frac{\partial H}{\partial \mathbf{u}_{\mathcal{K}}} = 2\rho \mathbf{u}_{\mathcal{K}}^* + \mathbf{B}_{\mathcal{K}}^T \mathbf{p}^* = 0. \tag{4}$$

This set of equations reduces to:

$$\begin{bmatrix} \dot{\mathbf{x}}^* \\ \dot{\mathbf{p}}^* \end{bmatrix} = \begin{bmatrix} \mathbf{A} & -(2\rho)^{-1}\mathbf{B}\mathbf{B}^T \\ -2\mathbf{I} & -\mathbf{A}^T \end{bmatrix} \begin{bmatrix} \mathbf{x}^* \\ \mathbf{p}^* \end{bmatrix} + \begin{bmatrix} \mathbf{0} \\ \mathbf{I} \end{bmatrix} 2\mathbf{x}_T \tag{5}$$

.

If we denote:

$$\tilde{\mathbf{A}} = \begin{bmatrix} \mathbf{A} & -(2\rho)^{-1}\mathbf{B}\mathbf{B}^T \\ -2\mathbf{I} & -\mathbf{A}^T \end{bmatrix} \tag{6}$$

$$\tilde{\mathbf{x}} = \begin{bmatrix} \mathbf{x}* \\ \mathbf{p}* \end{bmatrix} \tag{7}$$

$$\tilde{\mathbf{b}} = \begin{bmatrix} \mathbf{0} \\ \mathbf{I} \end{bmatrix} 2\mathbf{x}_T \tag{8}$$

then we can then write the reduced equation as:

$$\dot{\tilde{\mathbf{x}}} = \tilde{\mathbf{A}}\tilde{\mathbf{x}} + \tilde{\mathbf{b}} \tag{9}$$

which we can solve as:

$$\tilde{\mathbf{x}}(t) = e^{\tilde{\mathbf{A}}t}\tilde{\mathbf{x}}(0) + \int_0^t [e^{\mathbf{A}(t-\tau)}\tilde{\mathbf{b}}]d\tau \tag{10}$$

or, alternatively

$$\tilde{\mathbf{x}}(t) = e^{\tilde{\mathbf{A}}t}\tilde{\mathbf{x}}(0) + \mathbf{A}^{-1}(e^{\mathbf{A}t} - \mathbf{I})\tilde{\mathbf{b}}. \tag{11}$$

Then, substituting $t = T$, we arrive at:

$$\tilde{\mathbf{x}}(T) = e^{\tilde{\mathbf{A}}T}\tilde{\mathbf{x}}(0) + \mathbf{A}^{-1}(e^{\mathbf{A}T} - \mathbf{I})\tilde{\mathbf{b}}. \tag{12}$$

Let

$$\mathbf{c} = \mathbf{A}^{-1}(e^{\mathbf{A}T} - \mathbf{I})\tilde{\mathbf{b}}. \tag{13}$$

We can then write:

$$\begin{bmatrix} \mathbf{x}^*(T) \\ \mathbf{p}^*(T) \end{bmatrix} = \begin{bmatrix} \mathbf{E}_{11} & \mathbf{E}_{12} \\ \mathbf{E}_{21} & \mathbf{E}_{22} \end{bmatrix} \begin{bmatrix} \mathbf{x}^*(0) \\ \mathbf{p}^*(0) \end{bmatrix} + \begin{bmatrix} \mathbf{c}_1 \\ \mathbf{c}_2 \end{bmatrix} \tag{14}$$

Rewriting this, we get:

$$\mathbf{x}^*(T) = \mathbf{E}_{11}\mathbf{x}^*(0) + \mathbf{E}_{12}\mathbf{p}^*(0) + \mathbf{c}_1 \tag{15}$$

which can be rearranged to write:

$$\mathbf{p}^*(0) = \mathbf{E}_{12}^{-1}[\mathbf{x}^*(T) - \mathbf{E}_{11}\mathbf{x}^*(0) - \mathbf{c}_1] \tag{16}$$

Given $\mathbf{p}^*(0)$ and $\mathbf{x}_0$, we can then integrate $\tilde{\mathbf{x}}$ forward, thereby obtaining $\mathbf{x}_T$ from which we subsequently obtain the optimal inputs, $\mathbf{u}^*_{\mathcal{K}}$.

Note, in this derivation of the optimal inputs, we need only specify the free parameter ρ and the boundary conditions, namely $\mathbf{x}_T$ and $\mathbf{x}_0$. Collectively, these variables determine the value of $\mathbf{p}^*(0)$. It is also worth noting that while some of the variables have clear physical interpretations (e.g. $(\mathbf{x}_T - \mathbf{x})^T(\mathbf{x}_T - \mathbf{x})$ is the distance from the target state and $\mathbf{u}_{\mathcal{K}}^T\mathbf{u}_{\mathcal{K}}$ is related to the total energy) other variables do not. The additional variables that appear in this section are a consequence of the optimization technique that we used and come from the technique of Lagrange multipliers [2].

2 Justification for studying linear dynamics

In this paper we use linear control to drive the brain to and from specific states. The brain;s dynamics, however, are almost certainly non-linear. Therefore, it would be useful to at least consider the possibility of controlling non-linear models of brain activity. However, non-linear control is more complicated than linear control. To motivate our study linear control, we note that in some cases nonlinear systems can be well-approximated by linear systems. In computational and network neuroscience, for instance, linear systems have sometimes

been used as approximations of non-linear systems. For example, one study [?] used a linearization of the neural mass model described in earlier work (see [?, ?]), to show that it can generate predictions of the functional connectivity patterns between different brain regions/neural elements that are approximately as good as those obtained with the neural mass model, itself. Additionally (and as noted in [1]), if a linearized system is controllable then it implies that the non-linear system is locally controllable[?, ?]. Finally, linear models offer good approximations of their non-linear counterparts in the neighborhood or the operating point. Gain scheduling, for instance, uses multiple linear controllers to control a nonlinear system around operating points, toggling between different linear controllers when appropriate [?]. Thus, while brain dynamics are almost certainly non-linear, using linear models to investigate neural systems can still offer meaningful insight into the function and organization of neural systems.

3 Sensitivity of results to variation in parameters

The optimal control framework depends upon two free parameters (in addition to the initial and target states, $\mathbf{x}_0$ and $\mathbf{x}_T$, respectively) that must be selected by the user. The free parameter ρ determines the relative importance of the input amplitude to the distance from the target state. The other parameter, T, is the control horizon and specifies the time at which the system should be in state $\mathbf{x}_T$. In general, these parameters play a role in determining the optimal input signals. In this section, we explore how variation in these parameter influence some of the results presented in the main text.

3.1 Varying ρ

To test the robustness of our results to variation in the parameter ρ, we repeated the *full control* experiment (i.e. all nodes are control sites) for fifteen different values of ρ, logarithmically-spaced over the interval $[10^{-2}, 10^{5}]$ while fixing $T = 1$ (as in the main text). At each value of ρ, we obtained the energy input at each node for every possible transition (all combinations of initial and target states [4]). We compared these energy inputs at every pair of ρ values by calculating their Pearson correlation. If different ρ values corresponded to similar patterns of energy inputs then the correlation would be close to 1. Initially, we calculated the correlation of energies for the same control task (i.e. same pair of initial/target states) at each ρ; we subsequently averaged over these correlation values, so that for each pair of ρ we obtained a single average correlation coefficient.

The results of this exercise indicated that over a broad range of ρ values, the overall pattern of energy inputs changes very little (Figure S10). Over the range of ρ values that we explored, the smallest average correlation that obtained was 0.79 the largest 0.88 (the weakest correlation values was between $\rho = 10^{-2}$ and

$\rho = 10^5$). In general, this additional analysis supports the hypothesis that the results reported in the main text are representative over a large range of ρ.

3.2 Varying T

We repeated the above procedure varying the parameter T while fixing $\rho = 100$ (as in the main text). We varied T over the range $[10^{-3}, 10^1]$ in fifteen logarithmically-spaced points. This gave rise to, broadly speaking, two regimes: a region over short control horizons where the energy inputs are highly correlated with one another and a second regime over long control horizons where energy inputs are less similar to one another and also to the first regime (Figure S11). The value, $T = 1$, that we used in the main text, is located within the first regime, suggesting that, while the choice of T can influence the energy inputs, the value that we selected is representative of nearby parameter values.

4 Robustness to alternative definitions of functional systems

In the main text we defined initial and target states based on clusters/systems defined in [4]. To demonstrate that our results are robust to alternative definitions of functional systems, we repeated some of our results using the clusters described in [3], which have also been used in previous studies [1]. Using this alternative definition, we identified ten systems: auditory, cingulo-opercular, default mode, dorsal attention, fronto-parietal, other, somatosensory, sub-cortical, ventral attention, and visual systems. Using connectome data from a single subject, we tested whether we could predict the control energy in the *full control* experiment – i.e. where the control set included all brain regions. In general, our results were highly consistent with those reported in the main text (Figure S12). We observed that the energy for initial and target classes could be predicted with a high degree of certainty based on node degree ($r = 0.87$ and $r = -0.89$, $p < 0.05$). The ability to predict bulk node's energies was also predicted by node strength, but the correlation was not as strong ($r = 0.47$, $p < 0.05$). This supplementary result suggests that, irrespective of how we define our initial and target states, node strength is still a useful predictor of node's control energies.

5 Robustness to alternative parcellations and number of nodes

In the main text we described analyses conducted on a network of white-matter fiber pathways among $N = 129$ brain regions. In general, how a brain network's nodes are defined can influence its graph-theoretic properties [5]. To demonstrate that our results are robust to variations in node definition, we tested two alternative parcellations that give rise to different definitions of nodes: one that divided the brain into $N = 83$ nodes and another that resulted in $N = 234$

nodes. Using these node definitions, we constructed anatomical brain networks for a single representative subject using precisely the same techniques as described in the main text. Next, we used the optimal control framework to repeat the *full control* experiment – using the ten systems defined in [3] – and tested whether we could still predict node-level control energies based on node's strengths. As in the previous section, we observed excellent agreement with the results reported in the main text. Specifically, we found that with both 83-node and 234-node parcellation, the control energies of initial and target nodes were highly correlated with their respective strengths ($r = 0.9$ and $r = -0.91$ for the 83-node network and $r = 0.84$ and $r = -0.84$ for the 234-node network; Figure S13 A,B,D,E). Also in agreement with previous results, bulk node control energies were more difficult to predict, but nonetheless correlated with node strength ($r = 0.46$ and $r = 0.47$ for the 83-node and 234-node networks, respectively; Figure S13C,F). These supplemental results suggest that the results reported in the main text are robust to how we define brain network nodes.

References

[1] Roberto F Galán. On how network architecture determines the dominant patterns of spontaneous neural activity. *PLoS One*, 3(5):e2148, 2008.

[2] Shi Gu, Fabio Pasqualetti, Matthew Cieslak, Qawi K Telesford, B Yu Alfred, Ari E Kahn, John D Medaglia, Jean M Vettel, Michael B Miller, Scott T Grafton, et al. Controllability of structural brain networks. *Nature communications*, 6, 2015.

[3] Christopher J Honey, Rolf Kötter, Michael Breakspear, and Olaf Sporns. Network structure of cerebral cortex shapes functional connectivity on multiple time scales. *Proceedings of the National Academy of Sciences*, 104(24):10240–10245, 2007.

[4] CJ Honey, O Sporns, Leila Cammoun, Xavier Gigandet, Jean-Philippe Thiran, Reto Meuli, and Patric Hagmann. Predicting human resting-state functional connectivity from structural connectivity. *Proceedings of the National Academy of Sciences*, 106(6):2035–2040, 2009.

[5] Alberto Isidori. *Nonlinear control systems.* Springer Science & Business Media, 2013.

[6] Hassan K Khalil. Nonlinear systems, 3rd. *New Jewsey, Prentice Hall*, 9, 2002.

[7] Donald E Kirk. *Optimal control theory: an introduction.* Courier Corporation, 2012.

[8] Douglas J Leith and William E Leithead. Survey of gain-scheduling analysis and design. *International journal of control*, 73(11):1001–1025, 2000.

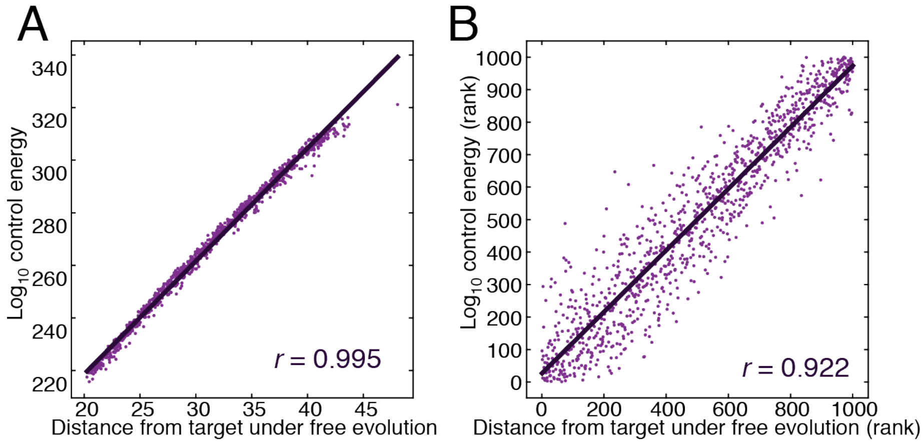

Figure S1: **Distance from target is correlated with control energy.** We generated 1000 random initial and target states and calculated both the total control energy, E, associated with the control task as well as the distance that the system would be from its target state given that it started in the initial state and evolved with no exogenous input, $\|v\|$. In calculating E we assumed that all nodes were directly controlled. *(A)* Scatterplot of the raw $\log_{10} E$ against $\|v\|$. *(B)* Scatterplot of ranked $\log_{10} E$ against $\|v\|$.

[9] Jonathan D Power, Alexander L Cohen, Steven M Nelson, Gagan S Wig, Kelly Anne Barnes, Jessica A Church, Alecia C Vogel, Timothy O Laumann, Fran M Miezin, Bradley L Schlaggar, et al. Functional network organization of the human brain. *Neuron*, 72(4):665–678, 2011.

[10] BT Thomas Yeo, Fenna M Krienen, Jorge Sepulcre, Mert R Sabuncu, Danial Lashkari, Marisa Hollinshead, Joshua L Roffman, Jordan W Smoller, Lilla Zöllei, Jonathan R Polimeni, et al. The organization of the human cerebral cortex estimated by intrinsic functional connectivity. *Journal of neurophysiology*, 106(3):1125–1165, 2011.

[11] Andrew Zalesky, Alex Fornito, Ian H Harding, Luca Cocchi, Murat Yücel, Christos Pantelis, and Edward T Bullmore. Whole-brain anatomical networks: does the choice of nodes matter? *Neuroimage*, 50(3):970–983, 2010.

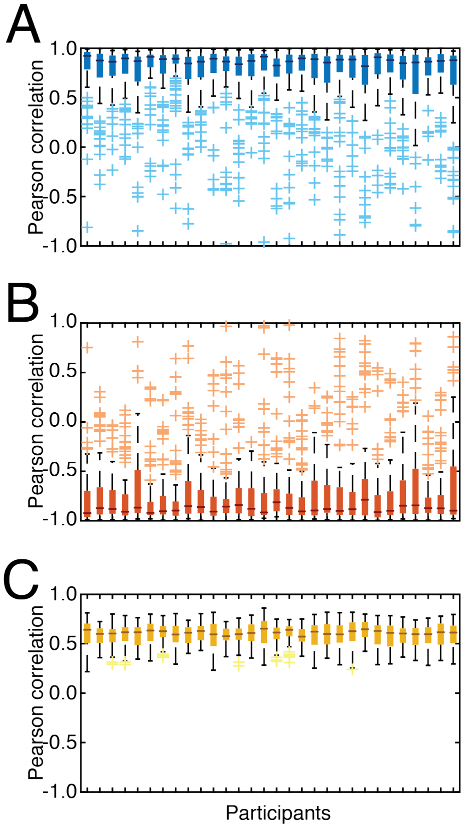

Figure S2: **Summary of full control across all participants.** In the main text we demonstrated that control energy was predicted by weighted degree (strength). Here we show that this same general pattern holds across all 30 participants. We show, here, for all three classes – *(A)* initial, *(B)* target, and *(C)* bulk – the distribution of correlation coefficients (logarithm weighted degree *versus* logarithm energy) obtained across all control tasks.

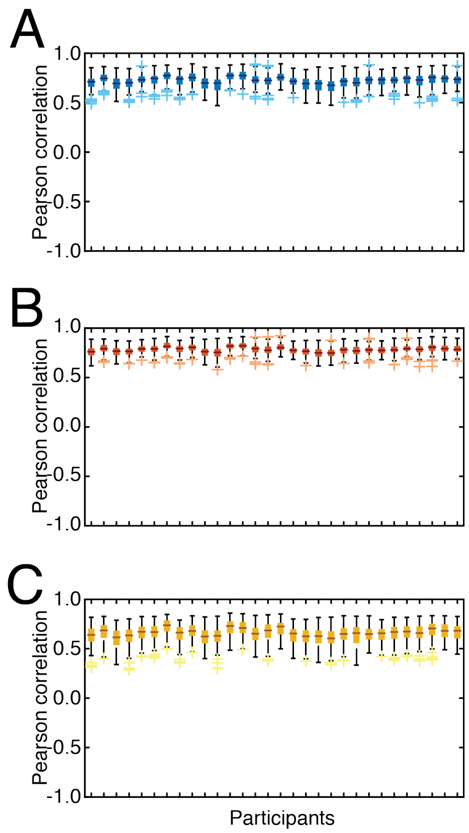

Figure S3: **Summary of single-region suppression across all participants.** In the main text we demonstrated that communicability between two regions predicted the extent to which either region compensated for the suppression of the other. We show, here, for all three classes – *(A)* initial, *(B)* target, and *(C)* bulk – the distribution of correlation coefficients (communicability *versus* percent change in energy) obtained across all control tasks.

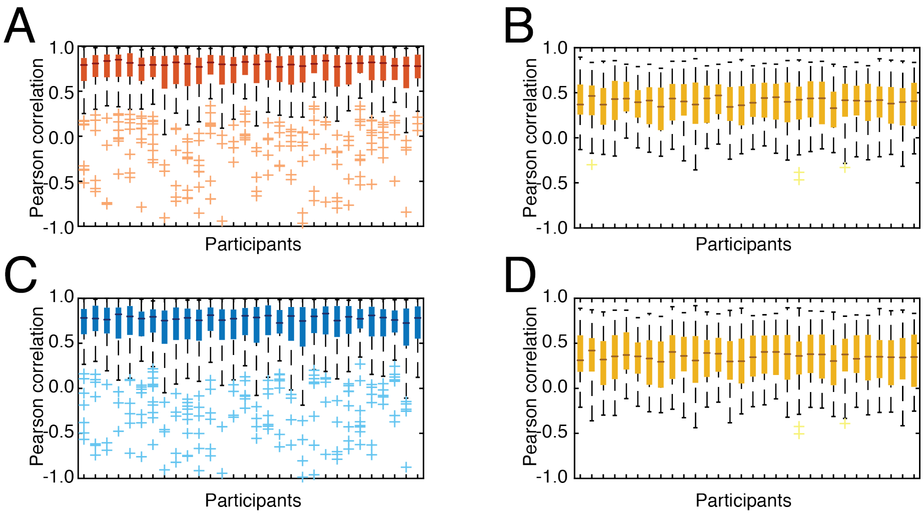

Figure S4: **Summary of initial or target class suppression across all participants.** In the main text we demonstrated that suppressing entire classes of brain regions (either initial or target classes) led to compensatory responses from the remaining brain regions. Moreover, we demonstrated that the percent change in energy of the remaining regions was closely predicted by their communicability to the suppressed class. Here, we show that this effect is consistent across all participants. Panels *(A)* and *(B)* show the distribution of correlation coefficients (communicability to initial class regions *versus* percent change in energy) obtained for target and bulk regions when we suppressed the initial class. Panels *(C)* and *(D)* show the same correlation coefficients but for initial and bulk classes when the target regions were suppressed.

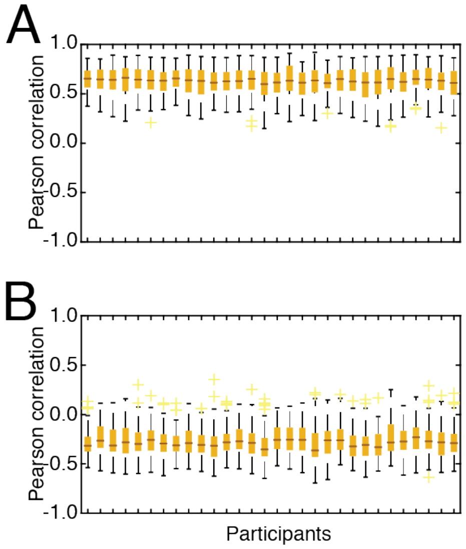

Figure S5: **Summary of initial and target class suppression across all participants.** In the main text we demonstrated that suppressing entire classes of brain regions (*both* initial or target classes) led to compensatory responses from the remaining brain regions. We showed that the energy associated with the remaining regions was proportional to their communicability to both suppressed classes and that their percent change in energy was related to how far their connected neighbors were from their desired state under free evolution. Here, we recapitulate those results for each participant. Panel *(A)* shows the distribution of correlation coefficients (energy *versus* communicability to initial and target classes) across all control tasks for each participant. Panel *(B)* shows the distribution of correlation coefficients (distance of neighbors from desired state *versus* percent change in energy).

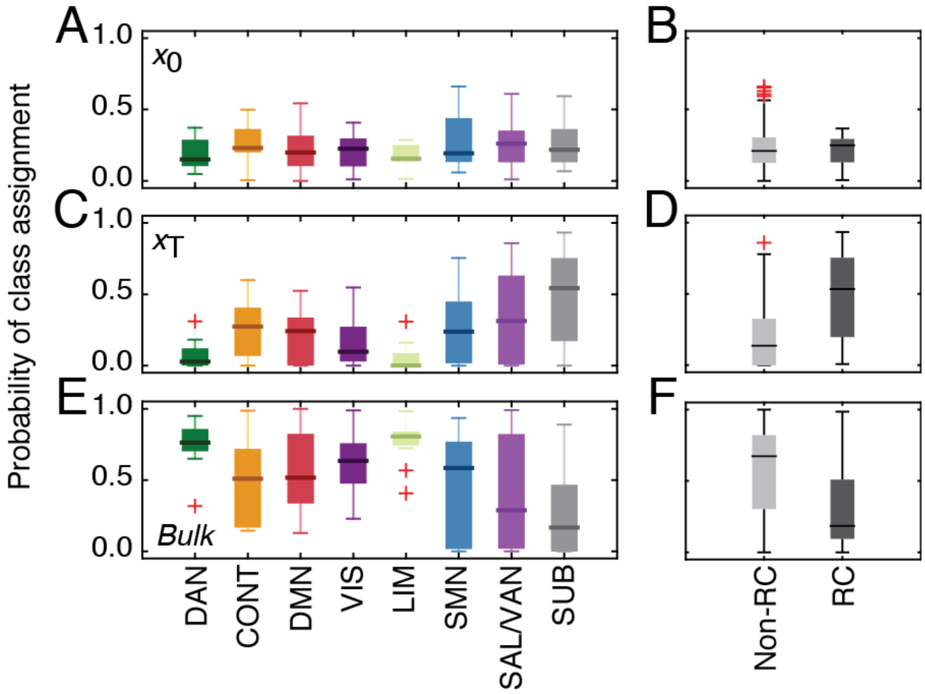

Figure S6: **Class assignments by brain system and rich club.** We aggregate class assignment probabilities according to brain system – panels *(A)*,*(C)*,*(E)* – and whether or those regions were assigned to the rich club at $k = 84$ – panels *(B)*,*(D)*,*(F)*. Each row represents a different node class: target ($\mathbf{x}_T$), initial ($\mathbf{x}_0$), and bulk classes. The probability of being assigned to the target class was statistically greater for rich club regions compared to non-rich club regions in 27/30 participants compared to 13/30 and 9/30 for bulk and initial classes, respectively ($p < 0.05$; Bonferroni corrected).

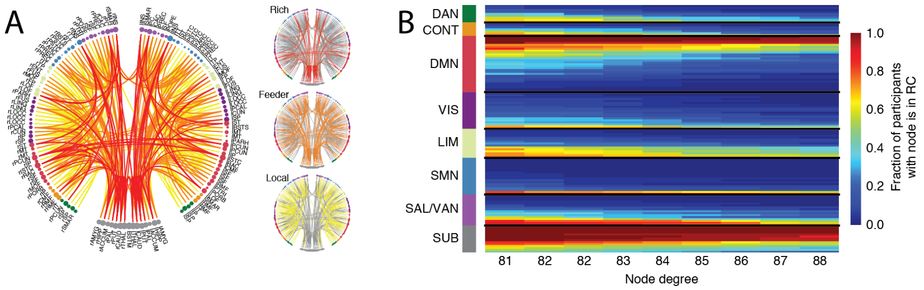

Figure S7: **Consistency of rich club.** In the main text we focus on the rich club defined at $k = 84$, shown in *(A)*. In *(B)* we show that the composition of the rich club over the range $k = 81$ to $k = 88$ is, generally, consistent. Each row represents a brain region and each column a different value of k. The color of each cell represents the fraction of participants for which a brain region was assigned to the rich club at a given k.

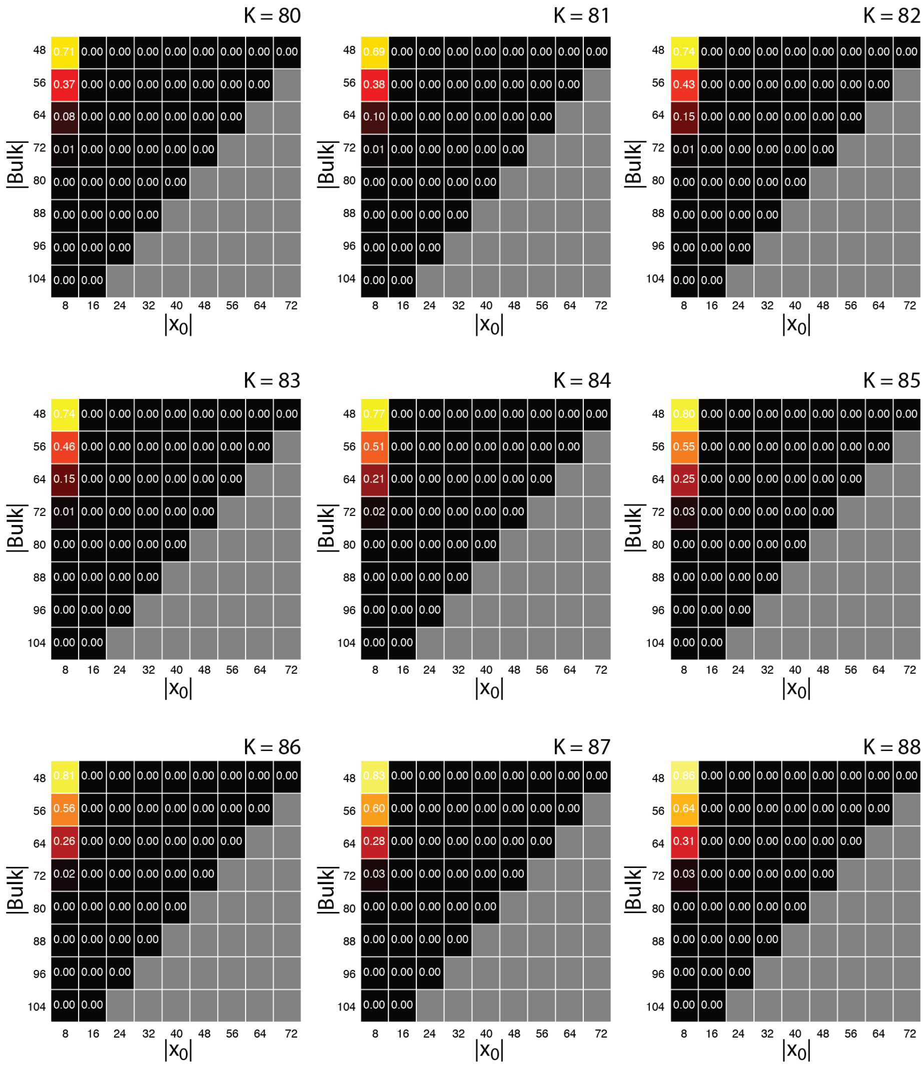

Figure S8: **Effect of rich club rewiring on energy.** In the main text we focus on the rich club defined at $k = 84$, demonstrating that when rich club connections were rewired the energy associated with transitioning from the optimal initial class to the optimal target class increased. Here we show the robustness of that result with respect to variation in the level at which the rich club was defined. Each plot shows a different rich club, ranging from $k = 80$ to $k = 88$. The y-axis shows the number of nodes assigned to the bulk class and the x-axis shows the number of nodes assigned to the initial class. Not shown is the number of target nodes, which can be calculated as $N - |\text{bulk}| - |\mathbf{x}_0|$. Gray cells correspond to class compositions that were not possible. The remaining cells display the probability that rewiring rich club connections will reduce energy, averaged across all participants.

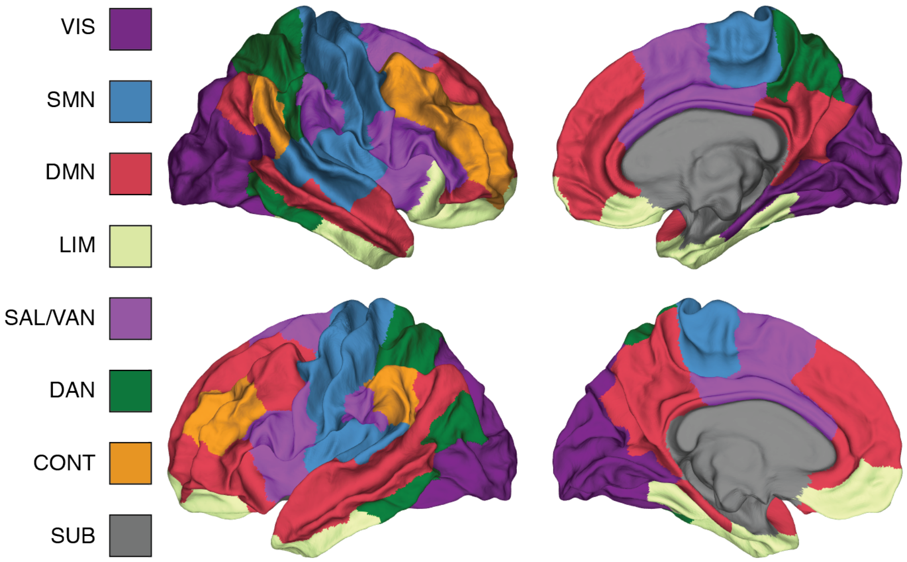

Figure S9: **Brain system assignments.** Topographic distributions of eight brain systems: Visual system (VIS; dark purple), somatomotor network (SMN; blue), default mode network (DMN; red), limbic system (LIM; cream), saliency/ventral attention network (SAL/VEN; light purple), dorsal attention network (DAN; green), control network (CONT; orange), and subcortex (SUB; slate).

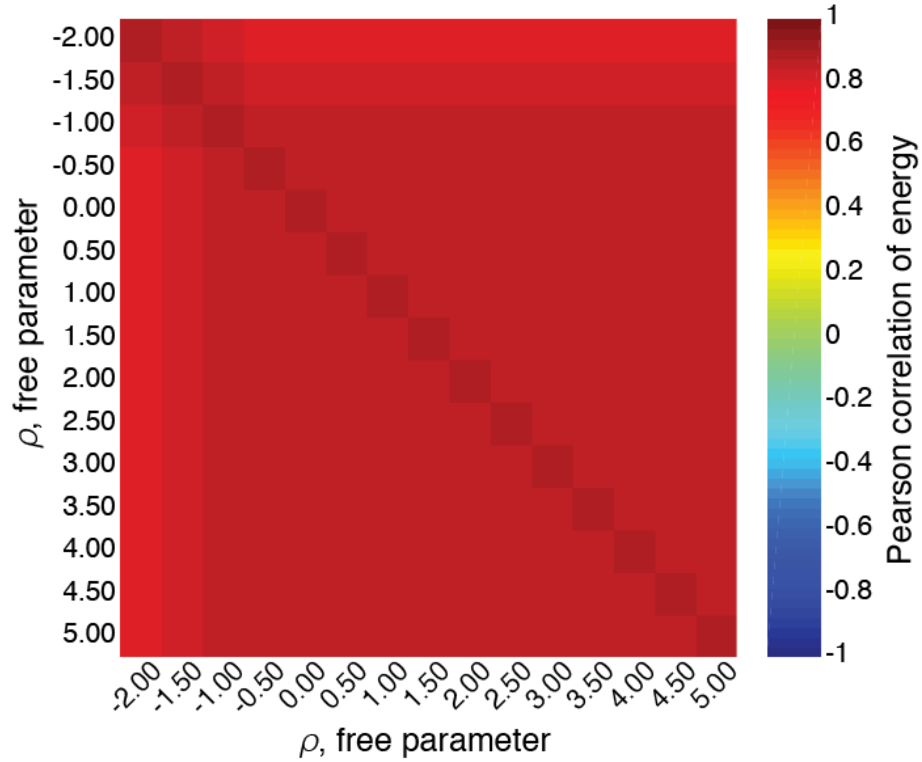

Figure S10: **Correlation of energy across different values of the parameter, ρ.** Matrix of Pearson correlation between node-wise energy inputs obtained using different values of the free parameter, ρ, and averaged over all possible transitions.

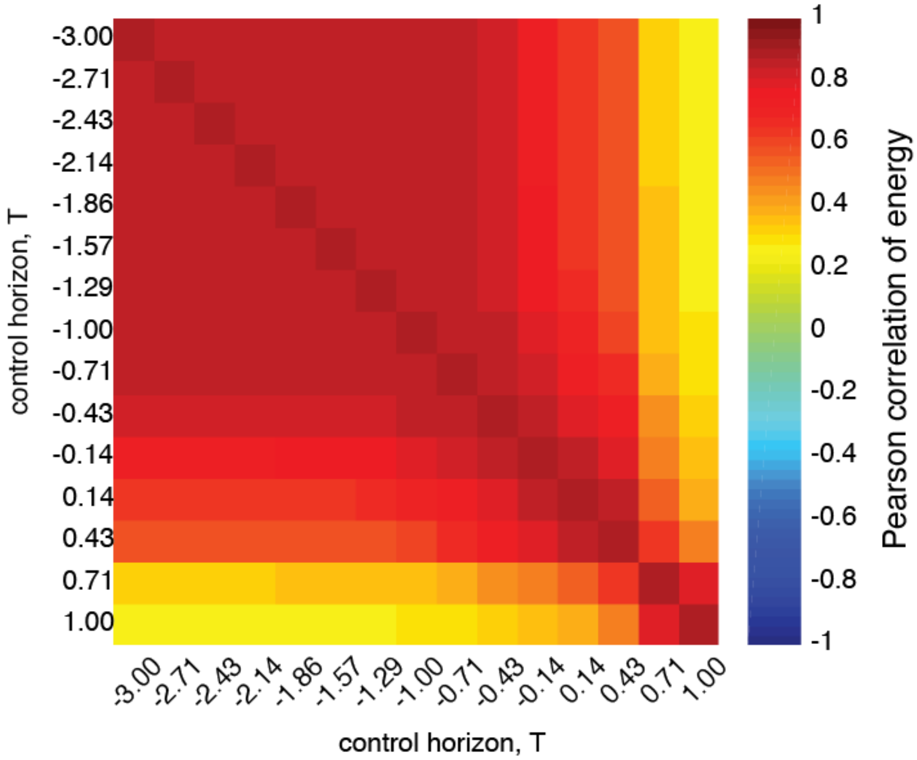

Figure S11: **Correlation of energy across different values of the parameter, T.** Matrix of Pearson correlation between node-wise energy inputs obtained using different values of the free parameter, T, and averaged over all possible transitions.

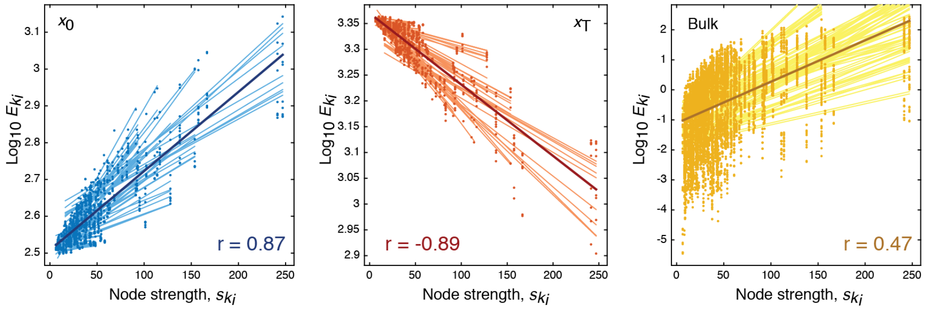

Figure S12: **Predicting energy with node strength using an alternative definition of functional systems.** We used a definition of functional systems different than that reported in the main text and considered all possible transitions. We show, here, that we can still do a good job in terms of predicting node-level control energies using node's strengths.

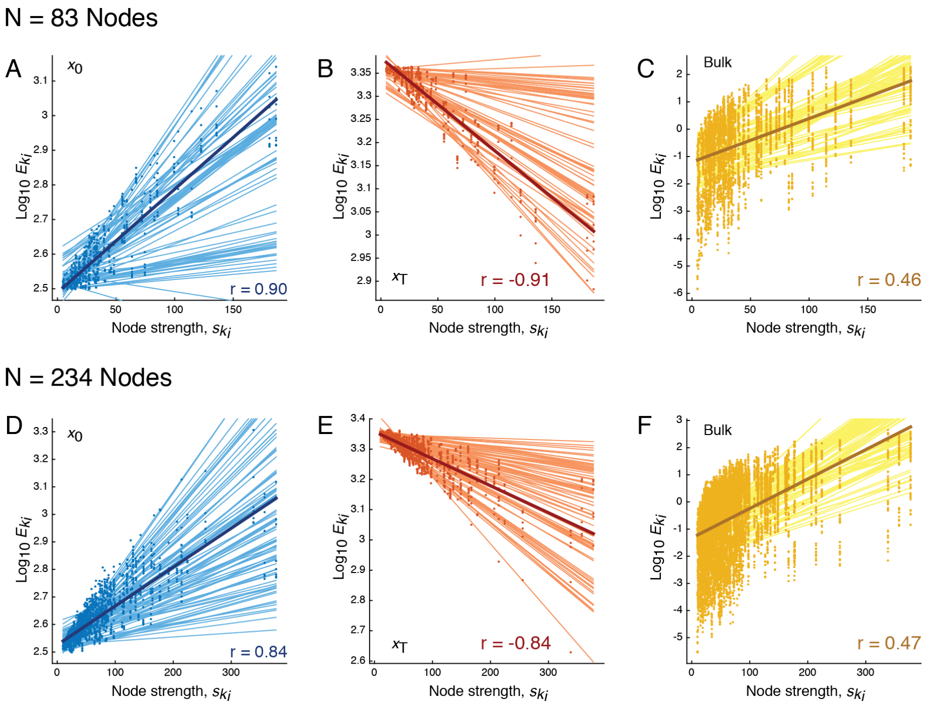

Figure S13: **Predicting energy with node strength using two alternative definitions of network nodes.** In panels (*A* - *C*) we show control energy versus node strength for the three classes – initial, target, and bulk – based on division of the brain into 83 nodes. In panels (*D* - *F*), we show similar plots but for a division of the brain into 234 nodes.
